# Acute Kidney Injury in Pediatric Acute SARS-CoV-2 Infection and Multisystem Inflammatory Syndrome in Children (MIS-C): Is There a Difference?

**DOI:** 10.3389/fped.2021.692256

**Published:** 2021-08-09

**Authors:** Manpreet K. Grewal, Melissa J. Gregory, Amrish Jain, Dunya Mohammad, Katherine Cashen, Jocelyn Y. Ang, Ronald L. Thomas, Rudolph P. Valentini

**Affiliations:** ^1^Division of Nephrology and Hypertension, Department of Pediatrics, Children's Hospital of Michigan, Detroit, MI, United States; ^2^Department of Pediatrics, Central Michigan University College of Medicine, Mount Pleasant, MI, United States; ^3^Division of Critical Care, Department of Pediatrics, Children's Hospital of Michigan, Detroit, MI, United States; ^4^Division of Pediatric Infectious Diseases, Department of Pediatrics, Children's Hospital of Michigan, Detroit, MI, United States; ^5^Department of Pediatrics, Wayne State University School of Medicine, Detroit, MI, United States

**Keywords:** COVID-19, multisystem inflammatory syndrome in children, risk factors, acute kidney injury, pathophysiology

## Abstract

**Objective:** To evaluate the prevalence and factors associated with the risk of acute kidney injury (AKI) in pediatric patients with severe acute respiratory syndrome coronavirus 2 (SARS-CoV-2) infection and multisystem inflammatory syndrome in children (MIS-C).

**Study Design:** We performed a retrospective chart review of 113 patients with SARS-CoV-2 infection with or without MIS-C admitted at Children's Hospital of Michigan (CHM) from March to August 2020. Patient demographic details, laboratory data, imaging studies, echocardiography reports, and treatment data were collected.

**Results:** Of the 92 patients included in the final analysis, 22 (24%) developed AKI with 8/22 (36%) developing stage 3 AKI. The prevalence of AKI was much higher in patients with MIS-C 15/28 (54%) vs. those with acute SARS-CoV-2 infection 7/64 (11%), (*p* < 0.001). Overall, when compared to patients without AKI, patients with AKI were older in age (11 vs. 6.5 years, *p* = 0.007), African American (86 vs. 58%, *p* = 0.028), had MIS-C diagnosis (68 vs. 19%, *p* < 0.001), required ICU admission (91 vs. 20%, *p* < 0.001), had cardiac dysfunction (63 vs. 16%, *p* < 0.001), required inotropic support (59 vs. 6%, *p* < 0.001) and had a greater elevation in inflammatory markers. In a multivariate analysis, requirement of inotropes [Odds Ratio (OR)−22.8, *p* < 0.001], African American race (OR-8.8, *p* = 0.023) and MIS-C diagnosis (OR-5.3, *p* = 0.013) were the most significant predictors for AKI. All patients had recovery of kidney function, and none required kidney replacement therapy.

**Conclusion:** Children with acute SARS-CoV-2 infection and MIS-C are at risk for AKI, with the risk being significantly greater with MIS-C. The pathogenesis of AKI in acute SARS-CoV-2 infection appears to be a combination of both renal hypo-perfusion and direct renal parenchymal damage whereas in MIS-C, the renal injury appears to be predominantly pre-renal from cardiac dysfunction and capillary leak from a hyperinflammatory state. These factors should be considered by clinicians caring for these children with a special focus on renal protective strategies to aid in recovery and prevent additional injury to this high-risk subgroup.

## Introduction

Acute kidney injury (AKI) in patients with severe acute respiratory syndrome coronavirus 2 (SARS-CoV-2) infection has been increasingly recognized and is associated with increased morbidity in adults. The prevalence of AKI in pediatric patients with SARS-CoV-2 infection is variable with reported rates ranging from 18 to 29% in hospitalized children (1–3); a higher prevalence has been reported in critically ill children approaching 44% according to a recent multi-center cross sectional analysis (4). A sub-set of patients with SARS-CoV-2 infection present with a hyper-inflammatory syndrome with multiorgan involvement called multisystem inflammatory syndrome in children (MIS-C), following acute coronavirus disease 2019 (COVID-19) infection (2). A variable proportion (5–73%) of MIS-C patients have been found to have AKI (2, 3). The pathophysiology of renal dysfunction in patients with SARS-CoV-2 infection is multifactorial including dehydration, poor cardiac output, cytokine storm, direct cytopathic effect of the virus on the renal tubular cells and use of nephrotoxic agents (5) whereas in patients presenting with MIS-C renal hypo-perfusion seems to be the major underlying factor for development of AKI (6). We conducted a retrospective chart review of pediatric patients with SARS-CoV-2 infection with or without MIS-C admitted to our hospital during the initial surge of the COVID-19 pandemic. We evaluated the prevalence of AKI and studied the factors associated with the risk of AKI in these patients. To our knowledge this is the largest single center cohort reporting the prevalence and factors associated with the risk of AKI in pediatric patients with SARS-CoV-2 infection and MIS-C.

## Materials and Methods

### Study Population

We performed a retrospective chart review of 113 patients who were admitted to Children's Hospital of Michigan (CHM) from March 2020 to August 2020. The study was approved by the Institutional Review Board at Wayne State University. A waiver of consent was granted.

### Inclusion Criteria

Study inclusion criteria were age <21 years, and either a—confirmed diagnosis of SARS-CoV-2 by a positive reverse transcriptase-polymerase chain reaction (RT-PCR) (with or without a positive SARS-CoV-2 antibody test), or SARS CoV-2 IgG antibody positive test only (with negative or unknown RT-PCR results). Patients with signs and symptoms of MIS-C with negative RT-PCR results and negative or unknown SARS CoV-2 IgG antibody test results were considered positive if an epidemiological link to patient with suspected or confirmed SARS-CoV-2 infection occurred within 4 weeks prior to onset of symptoms.

### Exclusion Criteria

Study exclusion criteria were patients who had a clinical presentation of MIS-C but did not meet the Centers for Disease Control and Prevention (CDC) case definition criteria. Patients positive for SARS-CoV-2 who were seen in the emergency department and discharged home the same day and patients hospitalized for symptoms unrelated to SARS-CoV-2 but with an incidental positive test were excluded from the final analysis.

### Case Definition

The case definition for MIS-C was based on the CDC's definition (7).

### Definitions

Kidney Disease: Improving Global Outcomes (KDIGO)-2012 criteria for defining and staging of AKI were used (8). AKI was defined by an increase in serum creatinine by ≥0.3 mg/dl within 48 h; OR increase in serum creatinine to ≥1.5 times baseline, which is known or presumed to have occurred within the prior 7 days. Staging of AKI was done as per KDIGO guidelines, and all three stages were included in the analysis. When baseline serum creatinine was not available, a presumed baseline eGFR (estimated glomerular filtration rate) of 120 ml/min/1.73 m^2^ was used to back-calculate the baseline serum creatinine using the modified Schwartz formula (9, 10). The urine output criteria were not used due to difficulty of data capture of urine output for every patient. Resolution of AKI was defined as eGFR > 100 ml/min/1.73 m^2^.

Echocardiography (ECHO) was used to assess cardiac dysfunction when clinically indicated and lowest ejection fraction (EF) recorded. Cardiac function was classified as normal (EF ≥ 55%); dysfunction was classified as mild (EF: 41–54%), moderate (EF: 31–40%), and severe (EF ≤ 30%) (11).

### Objectives

The primary objective of the study was to evaluate the prevalence of acute kidney injury (AKI) in pediatric patients with severe acute respiratory syndrome coronavirus 2 (SARS-CoV-2) infection and multisystem inflammatory syndrome in children (MIS-C). The secondary objectives were to study the severity, resolution and factors associated with risk of AKI.

### Data Collection

Data collection included patient demographic details [age, race, ethnicity, body mass index (BMI)], presenting symptoms, and underlying co-morbidities. Obesity was defined by a BMI > 95th percentile for age on the CDC growth charts for boys (12) and girls (13).

Hospitalization data collected included: level of care needed [intensive care unit (ICU)/inpatient floor], medications received and clinical support needs including inotropic support, mechanical ventilation, extracorporeal membrane oxygenation (ECMO), and kidney replacement therapy (KRT) and ECHO results. Laboratory data recorded included levels of blood urea nitrogen (BUN)/serum creatinine (at *baseline, admission, peak, and discharge)*, lowest serum albumin, peak values of c-reactive protein (CRP), creatine phosphokinase (CPK), d-dimer, troponin, brain natriuretic peptide (BNP), urinalysis, and urine culture (when available). Proteinuria on urinalysis was assessed in context of urine specific gravity (USG). Abnormal was defined as 1+ protein if USG < 1.020, 2+ protein if USG > 1.020 and < 1.035 abnormal and 3+ protein if USG > 1.035. Hematuria ≥ 2+ on urine dipstick or >5 RBC/hpf on urine microscopy was considered abnormal. Renal ultrasound, when available, was reviewed for increased echogenicity, impaired cortico-medullary differentiation, areas of infarct, or a size discrepancy of the kidneys. Information on home medications was collected.

Underlying co-morbidities incorporated into the analysis included: hypertension, diabetes mellitus type 1 (DM Type 1), diabetes mellitus type 2 (DM Type 2), transplant/immunosuppressive medications, cardiac disease (congenital or acquired heart disease), pulmonary disease (asthma/reactive airway disease/chronic lung disease), sickle cell disease, cancer, neurological disease (cerebral palsy/developmental delay, hydrocephalus/shunt, seizure disorder), and arthritis/vasculitis.

### Statistical Analysis

All descriptive statistics were summarized and displayed as median (range). Continuous variables with non-normal distribution were compared using the Mann-Whitney *U* test and categorical variables were compared using the Chi-square or the Fischer's exact test. A *p* < 0.05 was considered statistically significant. A multiple binary logistic regression model was performed with AKI as an outcome variable to ascertain the effects of potential predictor variables found significant on the univariate analysis and deemed clinically relevant.

A forward (conditional) selection was chosen to examine potential predictors with entry testing based on the significance of the score statistic, and removal testing based on the probability of a likelihood-ratio statistic based on conditional parameter estimates. The following risk factors were included in the model: MIS-C, African American race, requirement of inotropes and requirement of mechanical ventilation. Odds ratios (OR), *p* values and 95% confidence intervals (95% CI) were calculated. All analyses were performed using SPSS 27.0 software (Armonk, NY, IBM Corp).

## Results

We analyzed 113 patients seen at CHM from March 2020 to August 2020. Patients seen in the emergency department and discharged home the same day (*n* = 7) and patients hospitalized for symptoms unrelated to SARS-CoV-2 but with an incidental positive test (*n* = 14) were excluded from the final analysis. Our final analysis included 92 patients and of these, 22 (24%) developed AKI. The baseline characteristics of the patients are listed in Table 1. Median age of the entire cohort was 9 (range 0.1–21) years. Patients in the AKI cohort were significantly older with median age of 11 (range 1.8–21) years when compared to those in the non-AKI cohort where the median age was 6.5 (range 0.1–19) years (*p* = 0.007). There was a nearly equal distribution of males and females across both groups (59 and 49% males in AKI and non-AKI groups, respectively). Overall, 65% of the patients in the entire cohort were African American, but a higher percentage of patients in the AKI group were African American 19/22 (86%) when compared to those without AKI 41/70 (59%), (*p* = 0.028). Of note, no significant differences were noticed on the basis of ethnicity with only 2 patients of Hispanic origin, although there were nearly 14% patients with unknown ethnicity. Twelve of 13 patients with unknown ethnicity and none of the patients in the Hispanic group developed AKI. Medians of BMI across the two groups were similar with nearly 44% (9/22 in AKI group and 20/45 in non-AKI group) obese patients in both the groups.

**Table 1:** Baseline characteristics of children with SARS-CoV-2 infection with and without AKI.

	**All patients (*N* = 92)**	**AKI group (*N* = 22)**	**Non-AKI group (*N* = 70)**	***P*-value ^(&)^**
**Age group**				
<6 months	9 (10%)	0	9 (13%)	
6–23 months	13 (14%)	1 (5%)	12 (17%)	
2–4 years	10 (11%)	0	10 (14%)	
5–9 years	17 (19%)	7 (32%)	10 (14%)	
10–15 years	25 (27%)	7 (32%)	18 (26%)	
>15 years	18 (20%)	7 (32%)	11 (16%)	
**Median Age in years (Range)**	9 (0.1–21)	11 (1.8–21)	6.5 (0.1–19)	0.007
**GENDER**				0.389
Male	47 (51%)	13 (59%)	34 (49%)	
Female	45 (49%)	9 (41%)	36 (51%)	
**RACE**				0.028
African American	60 (65%)	19 (86%)	41 (59%)	
Caucasian	8 (9%)	2 (9%)	6 (9%)	
Unknown/Other	24 (26%)	1 (5%)	23 (33%)	
**ETHNICITY**				0.225
Hispanic	2 (2%)	0	2 (3%)	
Non- Hispanic	77 (84%)	21 (96%)	56 (80%)	
Unknown	13 (14%)	1 (5%)	12 (17%)	
**Median BMI (Range) in kg/m^2^^#^**	22.7 (12.9–54.6)	22.4 (13–54.6)	22.2 (13–50)	1.00
Non-Obese (%)	37 (56%)	12 (57%)	25 (56%)	0.904
Obese (%)	29 (44%)	9 (43%)	20 (44%)	
**Co-morbidities**	39 (43%)	13 (59%)	26 (37%)	0.069

& *Comparison between AKI (N = 22) and non-AKI (N = 70) patients for univariate analysis*.

# *BMI not calculated for 26 patients as patients were <24 months of age*.

**Figure 1:**
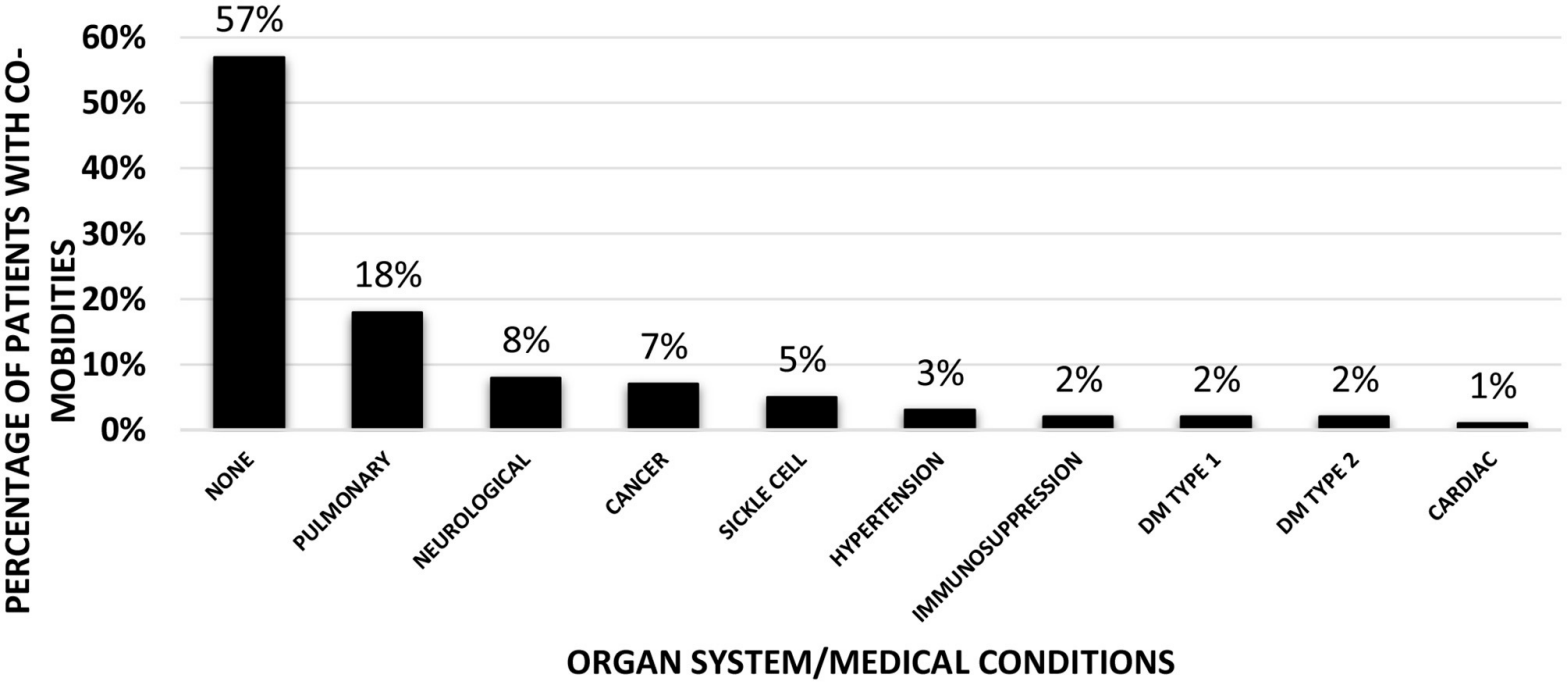
Co-morbidities of the patients in the entire cohort (*n* = 92). Bar graph showing percentages of patients with various underlying co-morbidities according to the organ system (Pulmonary/Neurological/Cardiac) or specific medical conditions (Cancer/Sickle cell anemia/Hypertension/Immunosuppression/Type 1 or 2 Diabetes Mellitus).

Overall, co-morbidities (Figure 1) were present in 43% of the entire cohort. Pulmonary (18%), neurological conditions (8%), cancer (7%), and sickle cell disease (5%) were the common comorbid conditions followed by small number of patients with hypertension (3%), immunosuppression (2%), type 1 (2%), and type 2 diabetes mellitus (2%), and cardiac conditions (1%). Nearly 60% (13/22) of patients in the AKI group had underlying co-morbidities as opposed to 37% (26/70) of patients in the non-AKI group (*p* = 0.069). Two patients in the non-AKI group were on angiotensin converting enzyme (ACE) inhibitors as home medications, whereas none of the patients in the AKI group were on ACE inhibitors or angiotensin receptor blockers.

**Figure 2:**
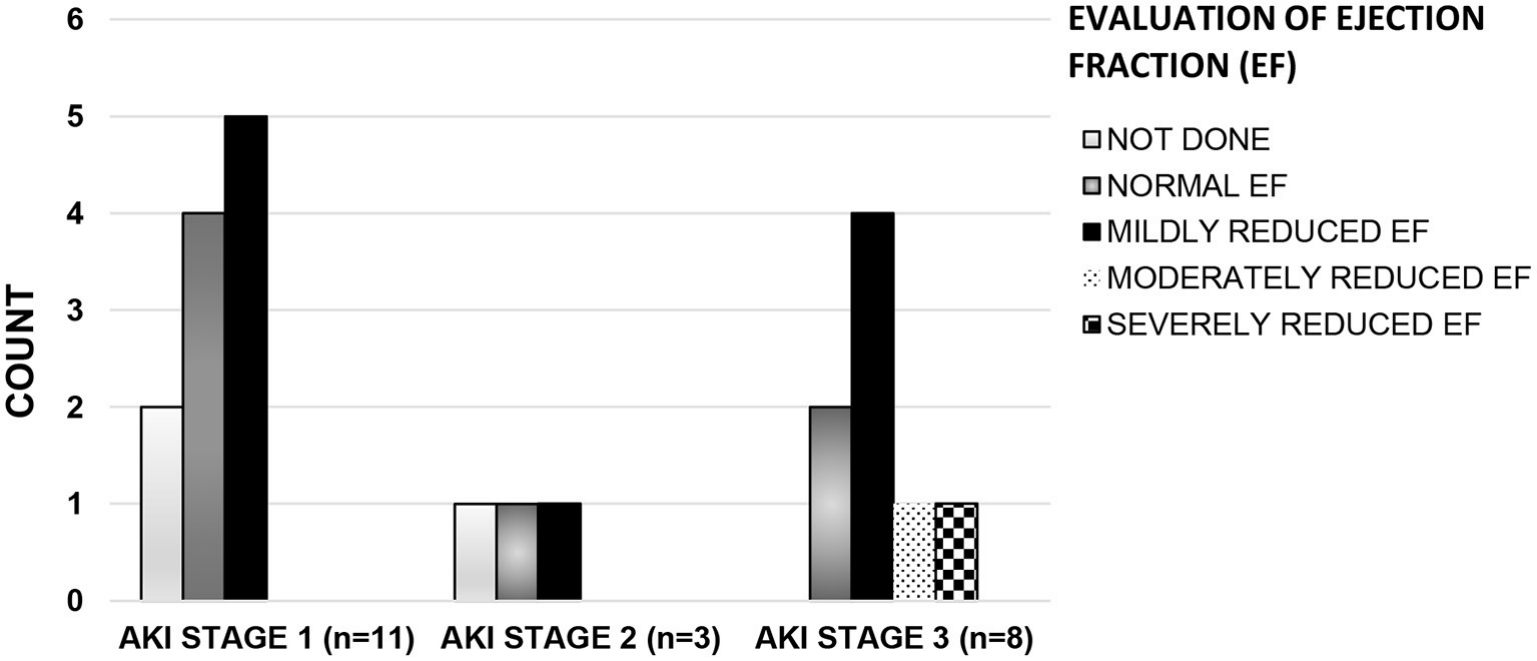
Left ventricular systolic function* by AKI stage^#^. Histogram showing the number of patients (Y axis) with varying degrees of cardiac dysfunction across all stages of AKI (X axis). X axis, Stages of AKI; Y-axis, Number of patients in each class of cardiac dysfunction. ^#^(AKI stage 1: serum creatinine 1.5–1.9 times baseline OR > 0.3 mg/dl increase within 48 h, stage 2: 2.0–2.9 times baseline, stage 3: 3.0 times baseline OR increase in serum creatinine to 4.0 mg/dl OR Initiation of kidney replacement therapy) (8). *[Left ventricular systolic function by Ejection Fraction (EF): Normal EF (EF ≥ 55%), Mildly reduced EF (EF: 41–54%), Moderately Reduced EF (EF: 31–40%), and severely reduced EF (EF ≤ 30%)] (11).

Evaluations and outcomes during hospitalization for all patients are presented in Table 2. In our entire cohort, 34/92 (37%) patients needed admission to the ICU and 20/34 (59%) patients had AKI. The median duration of ICU stay in patients with AKI was 7.5 days (range 1–25) while in non-AKI group this duration was 2.5 days (range 1–11), *p* = 0.025. Compared to patients without AKI, AKI patients had lower median serum albumin levels, greater median elevations in inflammatory markers and higher levels of markers of cardiac dysfunction. A urinalysis was available in 62 patients. The urinalysis was abnormal (variable degrees of hematuria, proteinuria, pyuria, or a combination of all three) in 47% (8/17) of the 17 patients tested in the AKI group, and in 13% (6/45) of the 45 patients tested in the non-AKI group (*p* = 0.005). Overall, 10 patients underwent a renal ultrasound examination. Three patients in AKI group had increased renal parenchymal echogenicity and two had normal renal ultrasound exams. Among the five patients in the non-AKI group who had a renal ultrasound, four were normal and the fifth patient had a wedge- shaped perfusion defect in the lower pole of the left kidney which resolved on repeat imaging 1 month later. A greater proportion (13/22) of AKI patients needed inotropic support compared to the non-AKI group (4/70) (*p* ≤ 0.001). Similarly, more patients in the AKI group (7/22) were mechanically ventilated compared to the non-AKI group (7/70) (*p* = 0.013). Fifteen (68%) of the 22 patients in the AKI group and 36/70 (51%) patients in the non-AKI group received nephrotoxic medications. An ECHO to evaluate cardiac dysfunction was available in only 38 patients (19 in AKI group and 19 in non-AKI group) in the entire cohort; 12 of 19 patients (63%) in the AKI group had an abnormal ECHO as compared to only 3 of 19 (16%) patients in non-AKI group (*p* ≤ 0.001). Severity of kidney dysfunction in patients with AKI, did not correlate with degree of cardiac dysfunction (chi-square = 0.655). Of the nineteen patients in the AKI group for whom ECHO results were available, ten patients had only mild decrease in ejection fraction but had varying degree of kidney dysfunction (Figure 2). No patient died in either group.

**Table 2:** Evaluation and outcomes during hospitalization.

	**All Patients (*N* = 92)**	**AKI group (*N* = 22)**	**Non-AKI group (*N* = 70)**	**P value ^(&)^**
**Patients with MIS-C**	28 (30%)	15 (68%)	13 (19%)	<0.001
**Median duration of hospital stay in days (Range)**	3 (1–43)	9.5 (1–43)	3 (1–16)	<0.001
**Patients admitted to the ICU (%)**	34 (37)	20 (91)	14 (20)	<0.001
**Median duration of ICU stay in days for patients admitted to the ICU (Range)**	4.5 (1–25)	7.5 (1–25)	2.5 (1–11)	0.025
**ECMO**	2 (2%)	2 (9%)	0	0.011
**Median peak serum creatinine (Range) (mg/dL)**	0.60 (0.2–4.25)	1.1 (0.55–4.25)	0.45 (0.2–0.9)	<0.001
**Median lowest serum albumin (Range) (g/dL)**	3.5 (1.7–6.0)	2.5 (1.7–5.0)	3.8 (2.3–6.0)	<0.001
**Median peak CRP (Range) (mg/L)**	114 (5–450)	276 (37–450)	69 (5–348)	<0.001
**Median peak CPK (Range) (U/L)**	127 (21–12149)	282 (21–12149)	122 (32–315)	0.213
**Median d-dimer (Range) (mg/L)**	3 (0.23–35)	5.15 (1.7–22)	1.82 (0.23–35)	0.002
**Median fibrinogen (Range) (mg/dL)**	595 (189–860)	627 (362–860)	469 (189–860)	0.025
**Median peak BNP (Range) (pg/mL)**	491 (16–5277)	2221 (491–5277)	90 (16–1396)	<0.001
**Median troponin (Range) (ng/L)**	40 (3–5880)	425 (3–5880)	11 (3–168)	<0.001
**Abnormal Urinalysis^e^**	14 (23%)	8 (47%)	6 (13%)	0.005
**Inotropic support**	17 (19%)	13 (59%)	4 (6%)	<0.001
**Mechanical Ventilation**	14 (15%)	7 (32%)	7 (10%)	0.013
**Nephrotoxic Medications Received^@^**	51 (55%)	15 (68%)	36 (51%)	0.167
**ECHO^∞^**				<0.001
Normal EF (≥55%)	23 (60%)	7 (37%)	16 (84%)	
Mildly decreased EF (41–54%)	13 (34%)	10 (53%)	3 (16%)	
Moderately decreased EF (31–40%)	1 (1.5%)	1 (5%)	0	
Severely decreased EF (≤ 30%)	1 (1.5%)	1 (5%)	0	
**Final Outcome**				
Death	0	0	0	
Discharge Home	92 (100%)	22	70	

& *Comparison between AKI (N = 22) and non-AKI (N = 70) patients for univariate analysis*.

e *N = 62 for overall cohort, N = 17 for AKI group and N = 45 for non-AKI group*.

@ *Nephrotoxic medications included vancomycin, acyclovir, and ibuprofen*.

∞ *N = 38 for overall cohort, N = 19 for AKI group, and N = 19 for non-AKI group*.

*MIS-C, Multisystem Inflammatory Syndrome in Children; CRP, C-Reactive Protein; CPK, creatine phosphokinase; BNP, brain natriuretic peptide; ECHO, Echocardiography; ECMO, extracorporeal membrane oxygenation; EF, Ejection Fraction*.

**Figure 3:**
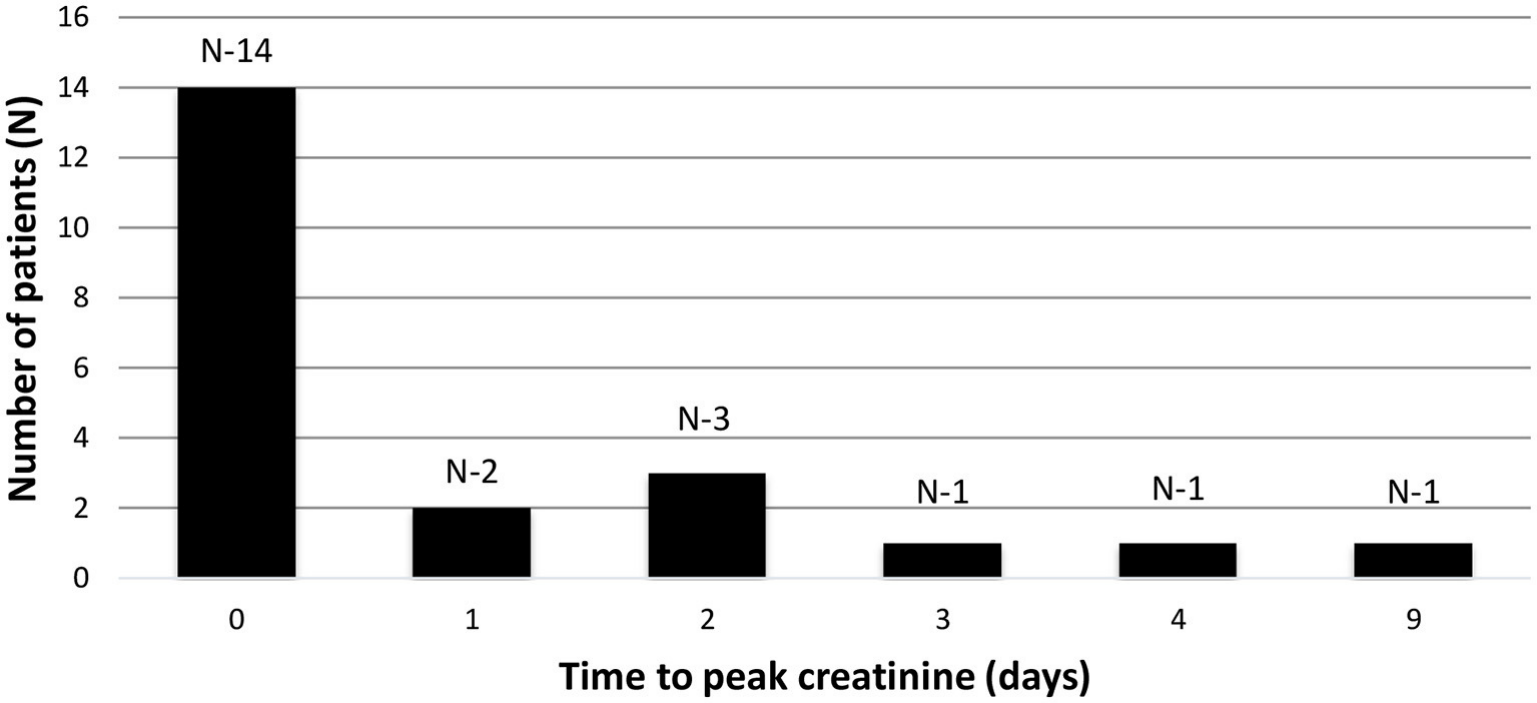
Time to peak creatinine. Bar graph showing the time to peak creatinine in days (X axis) and number of patients (y axis). Time zero depicts creatinine at admission or within first 24 h of admission.

In the AKI group, the time to reach peak creatinine ranged from 0 to 9 days and 63% (14/22) of the patients had AKI at presentation or developed it within the first 24 h of hospital admission i.e., day zero (Figure 3). The degree of kidney dysfunction was variable with 50% (*n* = 11) of the patients having stage 1 AKI; 14% (*n* = 3) with stage 2 and 36% (*n* = 8) patients with Stage 3 AKI (Table 3). The median (range) time to resolution of AKI was 4 (1–12) days and none of the patients required any form of KRT. At the time of discharge from the hospital, majority (18/22) of patients had complete resolution of AKI except for 4 patients who continued to have mild degree of kidney impairment with eGFR ranging between 60 and 90 ml/min/1.73 m^2^.

**Table 3:** Staging and outcomes of AKI patients.

**Staging and outcomes**	
**Stages of AKI^#^**	***N*** **(%)**
Stage 1	11 (50%)
Stage 2	3 (14%)
Stage 3	8 (36%)
**Median time to peak creatinine in days (Range)**	0 (0–9)
**Median time to resolution of AKI in days (Range)^$^**	4 (1–12)
**Patients with residual kidney impairment at the time of discharge (%)**	4/22 (18%)
**Kidney Replacement Therapy**	0

$ *Resolution of AKI: eGFR > 100 ml/min/1.73m2*.

# *Stage 1- Serum creatinine 1.5–1.9 times baseline OR > 0.3 mg/dl (> 26.5 μmol/l) increase*.

*Stage 2- Serum creatinine 2.0–2.9 times baseline*.

*Stage 3- Serum creatinine 3.0 times baseline OR increase in serum creatinine to > 4.0 mg/dl (> 353.6 μmol/l) OR Initiation of kidney replacement therapy OR, in patients <18 years, decrease in eGFR to <35 ml/min per 1.73 m2*.

A multivariate analysis revealed that requirement of inotropes (OR = 22.85, 95% CI: 4.17–125.1, *p* ≤ 0.001), African American race (OR = 8.86 and 95% CI: 1.35–57.82, *p* = 0.023) and MIS-C diagnosis (OR = 5.35 and 95% CI: 1.42–20.14, *p* = 0.013) were the strongest predictors of AKI in the entire cohort.

Twenty eight of the 92 patients presented with MIS-C and 64 patients had acute SARS-CoV-2 presentation. Fifteen (54%) of the 28 MIS-C patients had AKI compared to 7/78 (9%) patients with acute SARS-CoV-2 presentation (*p* < 0.001). A subgroup analysis between the MIS-C-AKI (*n* = 15) and acute SARS-CoV-2-AKI (*n* = 7) with regards to baseline characteristics and outcomes is presented in Table 4. Patients in the MIS-C-AKI group were significantly younger with a median age of 9 (range 1.8–17) years than patients in the acute SARS-CoV-2-AKI group who had a median age of 17 (range 10–21) years (*p* = 0.002). Both males and females were equally affected and the majority of the patients in both the groups were non-Hispanic, African Americans. MIS-C-AKI patients were predominantly non-obese 10/14 (71%) as opposed to acute SARS-CoV-2-AKI where the majority of the patients were obese 5/7 (71%); however, this difference did not reach statistical significance (*p* = 0.159). All patients in the acute SARS-CoV-2-AKI group had underlying co-morbidities, whereas 60% (9/15) patients in the MIS-C-AKI group had co-morbidities (*p* = 0.01). Median duration of hospital stay was 10 (range 4–23) days and 8 (range 1–43) days in the MIS-C-AKI vs. acute SARS-CoV-2-AKI groups (*p* = 1.00), respectively. All patients in the MIS-C-AKI group needed admission to the ICU while in the acute SARS-CoV-2-AKI group, 71% patients needed ICU admission. Patients with MIS-C-AKI had a longer median duration of ICU stay: 8 days (range 2–21) as compared to acute SARS-CoV-2-AKI group where the duration of ICU stay was 5 (range 1–25) days (*p* = 0.210). Eleven of the 15 (73%) patients in the MIS-C-AKI group required inotropic support, five patients were mechanically ventilated, and two patients required ECMO. Eight of the 15 patients in the MIS-C-AKI group had Stage 3 AKI but none required any form of KRT. None of the patients died in either group.

**Table 4:** Comparison between MIS-C-AKI and Acute SARS-CoV-2-AKI patients.

	**MIS-C-AKI (*n* = 15)**	**Acute SARS-CoV-2-AKI (*n* = 7)**	***P*-value**
**Median age in years (Range)**	9 (1.8-17)	17 (10-21)	0.002
**GENDER**			0.648
Male	8 (53%)	5 (71%)	
Female	7 (47%)	2 (29%)	
**RACE**			1.000
AA	13 (87%)	6 (86%)	
Non-AA	2 (13%)	1 (14%)	
**ETHNICITY**			
Hispanic	0	0	
Non-Hispanic	15 (100%)	7 (100%)	
**^&^BMI in kg/m^2^**			0.159
Non obese	10 (71%)	2 (29%)	
Obese	4 (29%)	5 (71%)	
**Co-Morbidities^#^**	6 (40%)	7 (100%)	0.01
**Median duration of hospital stay in days (Range)**	10 (4–23)	8 (1–43)	0.535
**Patients admitted to the ICU (%)**	15 (100)	5 (71)	0.09
**Median duration of ICU stay in days for patients admitted to the ICU (Range)**	8 (2–21)	5 (1–25)	0.210
**Median peak serum creatinine (Range) (mg/dL)**	1.11 (0.55–4.25)	1.11 (0.67–1.17)	0.574
**Median lowest serum albumin (Range) (g/dL)**	2.5 (1.7–5.0)	2.7 (2.4–4.4)	0.230
**Median peak CRP (Range) (mg/L)**	284 (37.5–450)	269 (129–431)	0.900
**Median peak CPK (Range) (U/L)**	194.5 (30–1386)	1074 (21–12149)	0.536
**Median d-dimer (Range) (mg/L)**	5.24 (2.48–22)	2.87 (1.69–7.16)	0.197
**Median fibrinogen (Range) (mg/dL)**	607 (362–860)	734.5 (689–780)	0.417
**Median peak BNP (Range) (pg/mL)**	2380 (661–5277)	491 (491)	0.200
**Median troponin (Range) (ng/L)**	534 (15–5880)	120 (3–448)	0.239
**Abnormal Urinalysis** ^**e**^	6 (43%)	2 (67%)	0.576
**Nephrotoxic medications received^@^**	12 (80%)	3 (42%)	0.08
**ECMO**	2 (13%)	0	1.00
**Inotropic Support**	11 (73%)	2 (29%)	0.074
**Mechanical Ventilation**	5 (33%)	2 (29%)	1.00
**ECHO**^^∞^^ Normal EF (≥ 55%), Mildly decreased EF (41-54%) Moderately Decreased EF (31-40%) Severely Decreased EF (≤ 30%)	5 (33%) 8 (53%) 1 (7%) 1 (7%)	2 (50%) 2 (50%) 0 0	0.602

& *BMI not calculated for one patient in MIS-C-AKI group due to age <24 months*.

# *Co-Morbidities as defined in Methods*.

*€ N = 14 for MIS-C-AKI group and N = 3 for Acute SARS-CoV-2-AKI group*.

@ *Nephrotoxic medications included vancomycin, acyclovir, and ibuprofen*.

∞ *N = 15 for MIS-C-AKI group and N = 4 for Acute SARS-CoV-2-AKI group MIS-C, Multisystem Inflammatory Syndrome in Children; CRP, C-Reactive Protein; CPK, creatine phosphokinase; BNP, brain natriuretic peptide; ECHO, Echocardiography; ECMO, extracorporeal membrane oxygenation; EF, Ejection Fraction*.

Among laboratory data, both groups had similar peak creatinine, serum albumin, fibrinogen, and peak CRP but patients with MIS-C-AKI had a greater elevation in D-dimer, peak BNP, and troponins. In 57% of the MIS-C-AKI patients the urinalysis was normal whereas two of the three (67%) available urinalyses in the acute SARS-CoV-2-AKI group were abnormal. Twelve (80%) of the 15 patients in MIS-C-AKI group and 3/7 (42%) patients in the acute SARS-CoV-2-AKI group received nephrotoxic medications (*p* = 0.08). All 15 patients in the MIS-C-AKI group had evaluation of cardiac status by an ECHO while 4/7 patients in the acute SARS-CoV-2-AKI group had an ECHO done. Ten of the 15 ECHO were abnormal in MIS-C-AKI group vs. 2/4 in the acute SARS-CoV-2-AKI group (*p* = 0.602).

## Discussion

In this study, we present our findings of 92 pediatric patients hospitalized at Children's Hospital of Michigan during the initial surge of the COVD-19 pandemic between March 2020 and August 2020, with SARS-CoV-2 infection with or without MIS-C. Twenty-two of the 92 (24%) patients met the KDIGO criteria for AKI with 8/22 (36%) having stage 3 AKI. Twenty-eight (30%) of the 92 patients had MIS-C and 15/28 (54%) of these patients were in the AKI cohort (*n* = 22). Reported incidence of AKI in pediatric population has been variable with initial reports from Wuhan, China suggesting a very low incidence (<1%) of AKI in critically ill patients with SARS-CoV-2 infection (14) to a single center study from UK, where Stewart et al. reported AKI in nearly 29% of their patient population (3). The reported prevalence of AKI in pediatric patients admitted to ICUs has also been variable with the Critical Coronavirus and Kids Epidemiologic study (CAKE study) reporting AKI in 18% of patients (1) while a recent multi-center cross sectional analysis of 106 critically ill children admitted to ICUs reported a much higher prevalence of AKI at nearly 44% (4). In our cohort, as well, 20/34 (59%) patients admitted to the ICU had AKI. Our results are similar to those of the above-mentioned studies (1, 3, 4) with a prevalence of 24% in the overall cohort and 59% in the patients admitted to ICU.

The majority of our patients with AKI were African American and older in age with no gender predisposition. African American patients have been shown to have a greater propensity of AKI from any cause (15) and our study demonstrates that the same holds true for SARS-CoV-2 patients, as well. This was further established by the multivariate analysis which revealed a nearly 9 times higher odds of AKI in African American patients. Interestingly, obesity which has been described as a risk factor for severe SARS-CoV-2 infection was not a significant risk factor for AKI in our cohort although other underlying co-morbidities were more predominant in patients with AKI (16).

As for the pathogenesis of AKI, one must look at both pre-renal and intrinsic renal causes. To better assess the causes of AKI in our cohort, we used a combination of tools including ECHO, serum markers of hyperinflammatory state (CRP, CPK, d-dimer, fibrinogen, and BNP), urinalysis, and renal ultrasound examinations. Our multivariate analysis revealed that children requiring inotropes had the strongest association with AKI –supporting a pre-renal component in the pathogenesis of AKI. On the other hand, indicators of renal parenchymal injury (abnormal urinalysis in 47% and abnormal renal ultrasound in 60%) were present in a significant proportion of our AKI patients suggesting that pre-renal may not be the sole mechanism for kidney dysfunction in our patient cohort. Previous studies have also demonstrated that a variable degree of hematuria and proteinuria is seen in patients with AKI secondary to acute SARS-CoV-2 infection (17) further supporting the fact that some degree of renal parenchymal involvement may be seen in these patients. The renal tropism of the SARS-CoV-2 has been previously demonstrated in autopsy studies of the renal tissue in adult patients who died secondary to SARS-CoV-2 infection (18, 19). Multiple kidney-cell types in all age groups have been found to be enriched in the genes for receptors which have been commonly described to facilitate the SARS-CoV-2 infection including the angiotensin-converting enzyme 2 (ACE2), transmembrane serine protease 2 (TMPRSS2), and cathepsin L (CTSL) (18). Su et al. in the autopsy specimens of renal tissue in patients who died from SARS-CoV-2 infection demonstrated diffuse proximal tubular injury and electron microscopy showing clusters of coronavirus-like particles with distinctive spikes in the tubular epithelium and podocytes (19).

Nearly two-thirds of patients in the AKI group had MIS-C which is characterized by a hyper-inflammatory state with multi-organ involvement. Our data demonstrated a higher likelihood of AKI in children with MIS-C (15/28 = 54%) vs. primary SARS-CoV-2 infection (7/78 = 9.0%) (*p* < 0.001). Our multivariate analysis further revealed that having MIS-C increased the odds of having AKI by nearly 5 times. A subgroup analysis of AKI patients comparing patients with acute SARS-CoV-2-AKI vs. those with MIS-C-AKI, showed patients with MIS-C-AKI were much younger but no differences were observed in terms of race, gender, and BMI. The MIS-C-AKI patients had longer durations of ICU stay, required more inotropic support, and had higher markers of cardiac dysfunction as compared to acute SARS-CoV-2-AKI patients while the general markers of inflammation were similar in both groups. Nearly 57% (8/14) of patients in the MIS-C-AKI sub-group had a normal urinalysis suggesting that renal parenchymal damage (from acute tubular necrosis or direct parenchymal injury from SARS-CoV-2 infection) might be less important in the pathogenesis of AKI in this patient group. Although our analysis was limited due to small sample size and hence was underpowered to detect a statistically significant difference between the two groups, these findings suggest that a different mechanism of kidney injury may exist in children with MIS-C compared to children with acute SARS-CoV-2 infection. The MIS-C-AKI group with its hyperinflammatory state and predisposition to capillary leak syndrome demonstrated a reduced EF in 10/15 (67%) and a normal urinalysis in 8/14 (57%) of AKI patients; these findings of low cardiac output and absence of urinary abnormalities suggest a pre-renal pathogenesis in this sub-group. Despite the importance of prerenal factors, the fact that 43% of patients in the MIS-C-AKI had an abnormal urinalysis means that kidney dysfunction is probably multifactorial, including both pre-renal and renal parenchymal causes. Our hypothesis that pre-renal factors are important in MIS-C-AKI patients is supported by other studies where a combination of poor cardiac function and a hyper-inflammatory state leading to capillary leak and third spacing of fluid have been described (20–23).

It is known that cardiac dysfunction can occur in a majority of patients with MIS-C (1, 2). In our cohort of MIS-C patients evaluated with ECHO, 13/25 (56%) had a decreased ejection fraction. Further, of these 13 patients with decreased EF, 10 patients (77%) developed AKI. The degree of cardiac dysfunction was mild in 8 patients with only 2 others demonstrating advanced cardiac dysfunction. Interestingly, the increasing severity of kidney dysfunction did not correlate with decreasing cardiac function as the majority of the patients with stage 3 AKI had only a mild decrease in EF (Figure 2). Thus, the role of hyperinflammatory state leading to capillary leak and third spacing of fluid cannot be ignored and might be a contributing factor to the development of AKI in this subset of patients.

Our data further supports the available literature on AKI in pediatric SARS-CoV-2 patients, in that AKI is a common morbidity in these patients and is possibly a combination of both renal hypo-perfusion and direct renal parenchymal damage. In patients with MIS-C presentation, the kidney injury appears to be predominantly pre-renal in nature. Careful monitoring of kidney function should be performed in these patients and all patients should be evaluated with urinalysis for hematuria and proteinuria.

The limitations of this study are its retrospective nature which interfered with our ability to quantitate the urine output and fluid overload in our patients. As such, serial measurements of serum creatinine were the sole means by which to assess AKI in our study. Another limitation was the lack of a uniform diagnostic evaluation of patients from a renal perspective in that not all patients had a urinalysis and quantification of proteinuria by urine protein/creatinine ratio was not performed in any of our hospitalized patients. Further, lack of data on urine sodium, prohibited our ability to evaluate the fractional excretion of sodium in our patients which could have helped better elucidate which of our patients were experiencing pre-renal vs. intrinsic renal injury as the probable cause of AKI. Lastly, our multivariable analysis is exploratory in nature, as is the speculation regarding the etiology of AKI in these patients, thereby necessitating the need for larger multi-center studies to validate our findings.

In summary, AKI is a significant but manageable cause of morbidity in pediatric patients with acute SARS-CoV-2 infection and the post-acute, MIS-C presentation with an excellent recovery. In this study, we highlight important clinical factors associated with development of AKI and the possible underlying pathogenesis in pediatric patients with acute-SARS-CoV-2 infection vs. MIS-C. Awareness among physicians about the extent of renal involvement in this patient population will not only help in timely diagnosis but also in proper management including minimizing exposure to nephrotoxic agents, management of the hyper inflammatory state, and appropriate initial fluid resuscitation, all of which can prevent long term morbidity and mortality. The fluid resuscitation especially in patients with MIS-C, should include careful use of IV fluids keeping in mind that these patients may have varying degree of cardiac dysfunction, and this may be worsened by aggressive fluid resuscitation. Hence, a low threshold for ECHO and use of inotropic agents, especially in the MIS-C subgroup is of particular importance. Our study represents a single center experience, and we believe that further well-designed prospective multi-center studies are needed to fully elucidate the true prevalence and pathogenesis of AKI in this patient population.

## Data Availability Statement

The raw data supporting the conclusions of this article will be made available by the authors, without undue reservation.

## Ethics Statement

The studies involving human participants were reviewed and approved by Institutional Review Board at Wayne State University Detroit, MI. Written informed consent from the participants' legal guardian/next of kin was not required to participate in this study in accordance with the national legislation and the institutional requirements.

## Author Contributions

MKG, DM, and RV conceptualized and designed the study and drafted the manuscript. MKG and DM performed the data collection. MKG and RT performed the statistical analysis. MKG, AJ, and RT contributed to the interpretation of the data. RV, MJG, AJ, KC, and JA provided intellectual content of critical importance to the work described. All authors contributed to revisions and provided the final approval of the version to be published.

## Conflict of Interest

The authors declare that the research was conducted in the absence of any commercial or financial relationships that could be construed as a potential conflict of interest.

## Publisher's Note

All claims expressed in this article are solely those of the authors and do not necessarily represent those of their affiliated organizations, or those of the publisher, the editors and the reviewers. Any product that may be evaluated in this article, or claim that may be made by its manufacturer, is not guaranteed or endorsed by the publisher.
